# Hospital Meetings

**Published:** 1896-05-09

**Authors:** 


### HOSPITAL MEETINGS.

### CITY OF LONDON HOSPITAL FOR DISEASES OF THE CHEST.

Lord Wolverton presided at the forty-second festival dinner of the above hospital, which was held on the 5th inst. at the Whitehall Rooms, amongst those present being: Earl Cowley, Sir H. R. Beevor, Bart., M.P., Mr. M. M. Bhownaggree, C.I.E., M.P., Mr. H. Robertson, M.P., Mr. J. H. Allcroft, Mr. J. Hill, Mr. H. C. Burdett, and Mr. Morton Latham. A feature of the dinners of this hospital is the number of representatives of working men's societies who are invited, and who show by their presence the deep interest taken by the working classes of the neighbourhood in this hospital. Not only, however, do these societies show their interest by their presence but by a far more practical method, namely, by their subscriptions towards the collection at the dinner. The following list of donations shows this: Albert and Victoria Society, 200 guineas; Bethnal Green Working Men's Society, 150 guineas; Excelsior Philanthropic Society, 140 guineas; Mutual Friendly Aid Society, 80 guineas; St. James's Philanthropic Society, 70 guineas; Good Intent Aid Society, 50 guineas; and the Mile End New Town Society, Haggerston Hospital Society, North Bow Society, and East London Association, 30 guineas each. Another point which illustrates the way in which the hospital is appreciated in the neighbourhood was mentioned by the chairman. No less than £50 was found in coppers in the donation boxes during the past year. Despite this support, however, the receipts from anything like certain sources, *i.e.*, annual subscriptions and the Hospital Sunday and Saturday Funds, amounts to only £4,500, out of a total expenditure of £11,000. Towards the deficit of about £6,500 a year the festival dinner in 1895 contributed £1,897. Mr. Burdett pointed out that the facts given above show how deep and earnest is the conviction of the working men resident in the neighbourhood that this institution is a necessity, that it is ably managed, and that the work done at it is one which they can support. Mr. Burdett was sure that if these facts could only be brought home to the wealthy inhabitants of London the contributions at the dinner would be raised from £2,000 to £5,000.

### ROYAL NATIONAL HOSPITAL FOR CONSUMPTION.

Festival dinners in aid of the funds of this institution are the exception rather than the rule, for it is now nine years since the last one was held, the Duke of Cambridge presiding on that occasion. The authorities, however, this year decided to use this form of appeal to the charitable, and a dinner was held at the Whitehall Rooms on the 29th ult., under the presidency of the Attorney-General, at which about 100 friends and supporters of the hospital were present. The hospital, which is beautifully situated in the Undercliff of the Isle of Wight, consists at the present time of ten blocks of buildings, with accommodation for 134 patients. This accommodation is not nearly sufficient, indeed, there are always about 100 patients waiting for admission—waiting oftentimes until too late—and the board of management have for some little time been considering the advisability of erecting a further block in order to be able to more adequately cope with the applicants for admission. Sir Richard Webster expressed a strong hope that before the next festival dinner the funds for erecting this house would be forthcoming, and announced that the block is to be named after the late Prince Henry of Battenberg, who took a deep interest in the well-being of the hospital. This institution, although situated at Ventnor, is not merely a local or even a metropolitan charity, but is in every sense a national one; for although nearly 47 per cent. of the total number of patients received have come from London, yet there is hardly a county in England which does not yearly send its quota of patients, whilst patients come from Scotland and Ireland, and have been sent even from the Channel Islands. It therefore appeals to all parts of Great Britain not only on account of the excellent work done to the individual, but for the value received by almost every district in the kingdom. Sir Richard Webster, in proposing the toast of the evening, "Prosperity to the Hospital," mentioned that the late Lord Randolph Churchill was very much struck with the great benefit which was felt by persons afflicted with consumption after a sojourn in South Africa, and that Lord Randolph suggested to him on more than one occasion that a scheme should be organised for sending patients there. Looking at the question from a practical and from an exhaustive point of view, he (Sir Richard Webster) had come to the conclusion that no such scheme could possibly supply the place of the work which had been and was still being done at Ventnor, because their work was essentially one of prolonging useful lives, and enabling the bread-winner of the family to again take up the burden and responsibilities of life. The result of the dinner was that a sum of over £1,230 was collected, including 50 guineas from Her Majesty the Queen, three sums of 100 guineas, and one of £100.

### CITY ORTHOPÆDIC HOSPITAL.

The anniversary festival dinner of this institution was held at the Holborn Restaurant on the 29th ult, under the presidency of the Duke of Fife. His Grace, who was still suffering from the effects of his recent accident, in proposing the toast of the evening said that no work could be more useful or noble than that which rectified or diminished the deformities of the young. It was difficult to exaggerate the lamentable results which must ensue to a child if its partially deformed frame were allowed to develop, and render it a hopeless cripple. The cases treated by this hospital were those requiring long and patient coaxing, such as could not usually be obtained at a general hospital, some of the patients remaining over two years in the wards. Orthopædic hospitals were not a mere scientific fad, but a merciful discovery not sixty years old, by means of which cases of deformity in children, which were at one time considered absolutely incurable, could now by gentle and slow treatment be either radically cured or very greatly alleviated. This hospital, which was established forty-five years ago to extend the work of its predecessor, the Royal Orthopædic Hospital, had no endowment of any kind. When the lease fell in five years ago the management were compelled to purchase the freehold and adjoining premises at a cost of £9,000, of which £6,000 was still due. Owing to this burden of indebtedness the committee had never been able to make the adjoining premises available, and, therefore, a space for thirty additional cots remained unoccupied. Although the hospital was not strictly a children's hospital, most of the patients were little children of the destitute poor. Such patients were, of course, totally incapacitated for any active employment, and were, so to speak, millstones round the neck of their families, and a burden to the rest of the community. Many of these cases had been entirely cured and many more greatly alleviated, so that by means of the hospital a considerable number of bread-winners had been added to the working population of this country. The secretary, Mr. E. Dereuth, announced subscriptions and donations amounting to £1,020, including £100 from Messrs. L. and A. Abrahams, and thirty guineas from the chairman.

### THE LONDON LOCK HOSPITAL.

The annual general meeting of the London Lock Hospital was held at the Male Hospital, Dean Street, Soho, W., on the 30th ult., Mr. E. Parker Young in the chair. The greater part of the time of the meeting was devoted to a laborious discussion of the annual report, reaching at length an animated controversy on merely verbal alterations. Seeing that many of those present were men of business whose time is valuable, it seems to us that it would have been far better to have settled the points raised at a meeting of the committee, so that the ordinary business of the annual meeting need not have been hampered with trivial matters like these. The Chairman, in moving the adoption of the report, said that it was one of the most satisfactory reports ever issued by that hospital, for the debt with which the hospital had been burdened for years had now been reduced to about £300. They had two matters for regret, however: first that two of their wards had to be kept closed owing to want of funds, and secondly that they had many applicants waiting for admission whom they could deal with if funds were forthcoming to enable those two wards to be opened.

